# Accessing public healthcare in Oslo, Norway: the experiences of Thai immigrant masseuses

**DOI:** 10.1186/s12913-019-4560-9

**Published:** 2019-10-21

**Authors:** Naomi Tschirhart, Esperanza Diaz, Trygve Ottersen

**Affiliations:** 1 Oslo Group on Global Health Policy, Department of Community Medicine and Global Health and Centre for Global Health, Institute of Health and Society, Faculty of Medicine, University of Oslo, P.O. Box 1130, Blindern, 0318 Oslo, Norway; 2 Unit for Migrant Health, Division for Health Services, Norwegian Institute of Public Health, Oslo, Norway; 3 Department of Global Public Health and Primary Care, University of Bergen, Bergen, Norway; 4 Division for Health Services, Norwegian Institute of Public Health, P.O. Box 222, Skøyen, 0213 Oslo, Norway

**Keywords:** International migration, Immigrant, Healthcare access, Equity, Universal health coverage, Thai, Norway, Masseuses, General practitioner

## Abstract

**Background:**

Thai massage is a highly gendered and culturally specific occupation. Many female Thai masseuses migrate to Norway as marriage migrants and as such are entitled to the same public healthcare as Norwegian citizens. Additionally, anyone who is not fluent in Norwegian is entitled to have an interpreter provided by the public healthcare system. Norway and most other countries aspire to universal health coverage, but certain immigrant populations continue to experience difficulties accessing appropriate healthcare. This study examined healthcare access among Thai migrant masseuses in Oslo.

**Methods:**

Guided by access to healthcare theory, we conducted a qualitative exploratory study in 2018 with Thai women working as masseuses in Oslo, Norway. Through semi-structured in-depth interviews with 14 Thai women, we explored access to healthcare, health system navigation and care experiences. We analyzed the data using thematic analysis and grouped the information into themes relevant to healthcare access.

**Results:**

Participants did not perceive that their occupation limited their access to healthcare. Most of the barriers participants experienced when accessing care were related to persistent language challenges. Women who presented at healthcare facilities with their Norwegian spouse were rarely offered interpreters, despite their husband’s limited capacity to translate effectively. Cultural values inhibit women from demanding the interpretation services to which they are entitled. In seeking healthcare, women sought information about health services from their Thai network and relied on family members, friends and contacts to act as informal interpreters. Some addressed their healthcare needs through self-treatment using imported medication or sought healthcare abroad.

**Conclusions:**

Despite having the same entitlements to public healthcare as Norwegian citizens, Thai migrants experience difficulties accessing healthcare due to pervasive language barriers. A significant gap exists between the official policy that professional interpreters should be provided and the reality experienced by study participants. To improve communication and equitable access to healthcare for Thai immigrant women in Norway, health personnel should offer professional interpreters and not rely on Norwegian spouses to translate. Use of community health workers and outreach through Thai networks, may also improve Thai immigrants’ knowledge and ability to navigate the Norwegian healthcare system.

## Background

Globally 244 million or 3.3% of the world’s population are international migrants living outside of their country of birth [1]. In Norway, immigrants make up 14% of the population (746700) [2]. As country populations become increasingly diverse, health systems must adapt to provide timely, appropriate and immigrant friendly health services. At the same time, many countries are pursuing Universal Health Coverage (UHC), with the goal that everyone has access to quality health services without experiencing financial hardship from using the services [3]. A large body of literature has demonstrated challenges to access and use for various immigrant groups in many different health systems, including those purporting to have UHC [4]. The Norwegian health care system is among those that score high on UHC, but previous studies have shown challenges for certain immigrant populations, including immigrants from Thailand [5–7].

There is a growing Thai population in Norway, and in 2018 there were almost 20,000 Thai immigrants in the country [8]. Most Thai immigrants are women who come to Norway as family immigrants and while Thais make up a small percentage of Norway’s overall immigrant population, Thai women represent the largest group who have moved to Norway to marry Norwegian men with non-immigrant backgrounds [9, 10]. Female marriage immigrants in Norway immigrate through regular administrative channels and have the same entitlements to healthcare as Norwegian citizens, including access to a general practitioner (GP) and primary healthcare services. Entitlements to healthcare in Norway are linked to administrative status and individuals with temporary and permanent residency status can access the public healthcare system. If a marriage immigrant divorces and maintains residency they will continue to have the same entitlements. In addition, all persons in Norway who are not fluent in Norwegian or have communication barriers are entitled to have an interpreter provided free of charge by the health care system [11].

Initial studies have found that language difficulties make it hard to access health information and navigate the health system for Thai immigrant women in Norway and that they use primary healthcare services for mental health less than Norwegian women [6, 12]. A study from nearby Sweden reported that Thai immigrant women preferred to wait to see a doctor in Thailand [13]. Having a Norwegian husband sets most Thai immigrant women apart from labour immigrants and refugees, and it is often hypothesized that these women will be able to access services and integrate into Norwegian society through the support of their Norwegian partner and his network [6].

Thai massage is a highly gendered and culturally specific occupation and many of the masseuses working in Oslo’s Thai massage shops are females born in Thailand. With just about half a million residents, Norway’s capital is reportedly home to over fourty Thai massage shops often run by Thai entrepreneurs [14, 15]. Thai massage emphasizes stretching and massaging pressure points. Clients can book massage by the hour. Thai massage is a job that utilizes cultural knowledge from the immigrants’ home country and allows them to merge into the Norwegian economy with minimal language skills. Massage requires limited literacy and is thus accessible as an occupation for new immigrants. Individuals who take up employment in massage shops may be marginalized from the mainstream Norwegian economy due to limited fluency in Norwegian and no study to date has examined their access to healthcare. In studies from other countries outside of Norway, Thai masseuses have been identified as grey zone workers as in some cases they provide sexual services in addition to traditional massage [16–18]. Women who provide sexual services may be at greater risk for sexually transmitted infections and much of the literature on Thai masseuses focuses on public health risk [16, 17]. Even if they are not providing special services, Thai masseuses must navigate sexualized stereotypes about Thai women on a daily basis [19]. As migrants, Thai masseuses negotiate these negative perceptions while also facing difficulties finding culturally and linguistically appropriate healthcare [16]. A study from the United States showed that masseuses have particular challenges accessing healthcare including lack of information, language barriers, limited finances, lack of insurance and fear of clinicians [16]. However, internationally Thai masseuses’ care seeking strategies, access to healthcare and experiences with care provided remain understudied. Healthcare access challenges for migrants have been well documented in Europe but there remains a need for qualitative inquiries documenting how migrants themselves navigate political and social landscapes [20, 21]. In addition gender, work, migration status and cultural expectations influence healthcare access and there is value in expanding the inquiry to consider these factors [22]. Access occurs at the intersection of populations and health systems and refers to an individuals’ ability to utilize the health services that they need [23]. For true access to exist, services need to be available, affordable, and appropriate and individuals need to have the resources necessary to be able to utilize them. The act of chartering a pathway to healthcare can be described as health systems navigation.

The objective of our study was to examine healthcare access among Thai migrant masseuses in Oslo. In this article we ask, what factors influence Thai migrant masseuses’ access to Norwegian healthcare and what strategies do they use to address their health needs.

## Methods

We employed a qualitative approach informed by a feminist theoretical basis that acknowledges women’s own agency in actively engaging with their environment to navigate pathways to healthcare [24]. Women’s agency remains a “situated, embodied and relational phenomenon” which is inextricably linked to larger social, political and power structures [24]. Individual agency as connected to the larger social and political environment fits well with Levesque et al’s [23] conceptual model on access to healthcare which we used to frame our enquiry. Levesque et al. [23] position that access to healthcare occurs at the intersection of health systems and populations’ ability to utilize them. In employing a research agenda that emphasizes female migrants’ agency, we inquired about navigating pathways to care and strategies women utilize to gain access to healthcare.

**Fig. 1:**
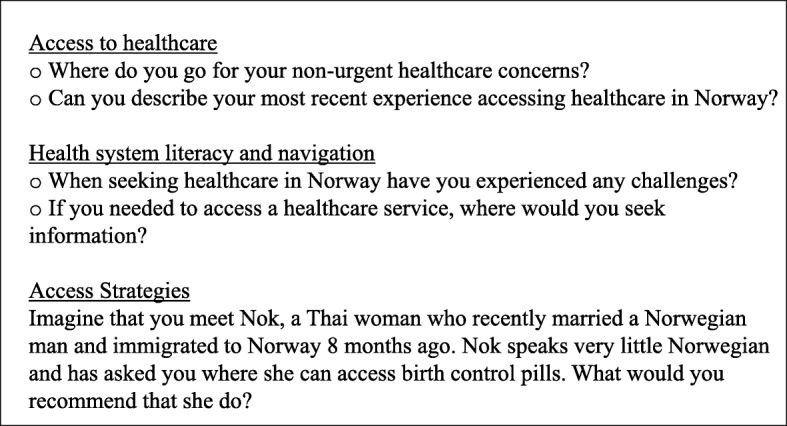
Abbreviated Interview Guide

We conducted semi-structuredin-depth interviews with 14 Thai women who were working as masseuses in Oslo, Norway. In collaboration with a local NGO, NT visited massage shops in Oslo, introduced our project in Thai language and recruited participants. Individuals who migrated to Norway, worked as a masseuse in Oslo in the last 12 months, were 18 or older, identified as female and spoke sufficient English or Thai were invited to participate. We systematically invited all individuals who fit the inclusion criteria and employed snowballing to recruit additional participants by inviting interviewees to share information about the study with their network. Participants provided consent to participate prior to the beginning of each interview. NT conducted 13 individual interviews with Thai masseuses during the fall of 2017 with the assistance of a Thai interpreter. One interview was completed in English. Using a semi-structured interview guide we explored access to healthcare and health system navigation. See Additional file 1 for the full interview guide and Fig. 1 for the abbreviated guide.

All interviews were audio recorded and later simultaneously transcribed and translated into English by the interpreter, a Thai native speaker who has graduate training in research methods. After each interview NT and the interpreter discussed the content and identified new themes or experiences. We reached data saturation with 14 participants, in that the last few participants did not contribute any new information related to the research inquiry. Upon reaching data saturation, we discontinued recruitment of participants. NT reviewed all of the transcripts, discussed segments of the translation with the interpreter as necessary, developed a coding frame and coded the data using NVivo software (version 11). NT applied thematic analysis to identify themes of interest based on our interview guide as well as new themes which emerged from the data [25]. We used the concept of intersectionality to analyze the results and to contextualize participants’ experiences. Throughout this article we use pseudonyms for participants’ names, present ages in 10 year ranges and have redacted or masked all personally identifying information. In recognizing participants’ intersectional experience, as Thai female transnational migrants, we utilize quotes to put forward information in their own words [26].

The first author, a Canadian migrant in Norway, has lived and conducted migration research in Thailand and speaks elementary Thai. Co-authors are a Spanish immigrant living in Norway and Norwegian respectively, with knowledge of migrant health in Norway and the Norwegian healthcare system.

## Results

### Demographic information

Of the 14 participants, seven immigrated directly from Thailand, six came from Sweden and one came from another EU country. More than half of participants [9] came from rural Thailand and all participants had initially come to Europe because of an intimate relationship with a male citizen in their destination country. At the time of the interviews, most of the participants were divorced or separated and 40 years of age or older (see Table 1). Participants had been in Norway for varying amounts of time. Education levels also varied, and almost half completed lower secondary school (grade 9 or below). Participants largely reported limited Norwegian language fluency, although some [4] spoke Swedish which is linguistically close to Norwegian.

**Table 1:** Characteristics of participants (*N = 14*)

Characteristics	N
Age	
< 34–39	3
40–45	4
> 45	7
Highest Level of Education	
< Grade 10	6
High school	5
Post secondary	3
Years in Norway	
< 2	5
3–10	4
> 10	5
Fluency in Norwegian	
Limited	3
Beginner	7
Intermediate	3
Not stated	1
Marital Status	
Married/Cohabitating	2
Separated/Divorced	9
Widowed	1
Single	2

### Factors that influence access to healthcare

Participants did not perceive that their occupation as masseuses limited their healthcare access. Masseuses could access testing for sexually transmitted infections and other reproductive health services from a public health outreach team that came to visit the massage shops and didn’t report any unmet reproductive health needs. Due to the physical nature of their work, some participants experienced musculoskeletal problems including joint and back pain but their occupation did not impact their ability to seek care. Several participants reported unmet mental health needs, but did not see their job as a barrier to care. Instead masseuses’ access to healthcare was affected by their knowledge of the Norwegian system, waiting time for appointments, language difficulties, positionality as a marriage migrant and cultural values.

#### Knowledge of the Norwegian healthcare system

Most of the respondents were knowledgeable about Norway’s registered general practitioner (GP) scheme, which is the basis of primary healthcare in the country, and over half had a GP. Overall, respondents were well informed about the GP’s role as a gatekeeper for the Norwegian system and the potential to get referrals for specialty care. The few who had not heard about the scheme had only been in the country for 2 years or less and several had a GP in a nearby Sweden.

Daw (50–59) who had only been in the country for 6 months was uncertain about the location of health care services, and how to navigate the system, “I do not know how to get to see a doctor. I do not know where are the doctor clinics, how I travel to see the doctors, how much the medical fees are.”

Overall participants viewed the Norwegian healthcare system in general as high quality and appreciated having a GP and affordable access to emergency care at the hospitals. Hom (50–59, 18 years in Norway) expressed, “Here the healthcare is good.” Participants explained that the Norwegian health care system differs from Thailand as in Norway access to medicine including antibiotics is more controlled and requires a doctors’ prescription comparative to Thailand where they can be directly purchased from a pharmacy. Despite this difference, participants reported being very satisfied with the quality of Norwegian healthcare and the overall organization of the system. While pleased with the quality of Norwegian healthcare, participants found it was difficult for them to access care.

Some participants did not know that they could request a free interpreter when seeking healthcare in Norway, while others had heard about the service but did not know how to request it. The entitlement was sometimes perceived to be only permissible in a very serious case like a surgery.

#### Waiting time for appointments

For some participants waiting time to get a doctors’ appointment with their GP through the public system influenced their decision to seek care for non-urgent health concerns. Long waiting times were associated with a reluctance to seek care.

“If you want to see a doctor, you have to make an appointment. Doctors would look when they are available. By the time you got to see doctors, you already recovered.” Kwang (40-49), 4 years in Norway

Communication difficulties in Norwegian, also contributed to longer waiting times for as women needed to seek assistance to book the appointment which lengthened the process.

#### Language difficulties

Participants identified language difficulties as their most persistent challenge when locating information about health services and accessing healthcare in Norway. Norwegian language capacity influenced women’s ability to locate official health service information, book appointments, utilize care and comprehend care providers’ instructions. The few participants with high level Norwegian or English had less difficulties accessing healthcare than those with limited fluency. While more recent arrivals had greater challenges navigating the healthcare system, language barriers created consistent navigation difficulties even among women who had been in the country for several years.

“My problem is the language. As I have to book the appointment, we call or book via the internet. This is a problem for people who do not have language proficiency (in Norwegian).” Isra (30-39), 3 years in Norway

Even among those with intermediate levels of Norwegian, the medical terms used by doctors made the information difficult to comprehend.

“The problem is about language. Sometime doctors say something that I do not understand. They used medical vocabulary. Even I could not understand it.” Hom (50-59), 18 years in Norway

In addition, one participant indicated that regional dialects spoken by healthcare staff were difficult to understand as she had only learned the Oslo dialect.

#### Positionality as a marriage migrant

Being married to a Norwegian citizen had important implications for participants’ access to healthcare, especially among women with limited fluency in Norwegian or English. It was sometimes assumed that the Norwegian partner would help facilitate their Thai wives’ utilization of the Norwegian healthcare system. Often, when participants presented at healthcare facilities with their Norwegian husband, they were not offered an interpreter and instead it was expected that their husband would translate. This caused difficulty as husbands often had insufficient linguistic capacity in Thai to be able to translate effectively.

“Most people who have a Norwegian husband will ask them to come (to the doctors’ office). But, some women do not understand their husband’s explanation really well because of the language.” Wattana (40-49), 15 years in Norway

In some cases, presenting with a Norwegian husband and experiencing communication difficulties, influenced women’s willingness to engage with the Norwegian healthcare system and their associated care seeking strategies.

“The reasons they (Thai masseuses) go to Thailand for operations, even those who have Norwegian husbands who accompany them to the hospital, is that when seeing doctors in Norway their husbands could not translate medical information about future treatments (given by Norwegian health care providers) effectively. Those Thai ladies could not really understand about the future medical operations.” Daw (50-59), 0.5 years in Norway

Having husbands act as de facto interpreters relies on the kindness or compassion of the individual and his willingness to assist his partner. In one case, a participant explained that her husband would assist her but expected an unspecified favour in return which could lead to an abusive situation.

“Mostly, if your husband accompanies you they would not offer or ask you about the translator service. For those people who meet good husbands, they are very fortunate. But for me, I met a bad husband. He will use the fact that I rely on him to be my translator with doctors to abuse me later. In some other cases, when I want him to do something for me, he will put up conditions for me to do something for him in exchange. It depends on who you meet.” Kwang (40-49), 4 years in Norway

Participants differentiated between good and bad husbands. Several participants had experienced physical, emotional or economic abuse within their past marriages and used the term “bad husbands” to describe their former partners.

#### Cultural values

One participant explained that cultural values constrict Thai immigrant women from demanding an interpreter or complaining about the care they receive.

“Yes, we need to request (a translator). It is only the policy that makes Norway look good. But in reality, you have to keep complaining to get a translator. Thais do not like to be demanding. This is a problem. Most Thai women do not like to talk too much, they will help themselves. I brought my own medicine, I will not beg for your help.” Sanit (40-49), 10 years in Norway

Being shy in addition to communication difficulties may lead to a reluctance to seek care, let alone request an interpreter.

“Some masseuses are afraid to see doctors because they are shy and face a language barrier.” Achara (40-49), 2 years in Norway

Taking an approach where one advocates for themselves and requests an interpreter was identified as undesirable. Self-reliance, was instead emphasized. A hesitancy to demand services from the government extends outside healthcare and into other social sectors.

“Frankly, most of Thai ladies who come here will not ask for financial assistance from the government, we will work.” Kannika (40-49), 17 years in Norway

### Strategies to access care for non-urgent health concerns in Norway

When experiencing non-urgent health concerns in Norway our participants either employed strategies to utilize the Norwegian healthcare system or sought other solutions including self-treatment. Some also combined the use of services within and outside of the Norwegian system.

#### Strategies to utilize the Norwegian healthcare system

Women employed a network approach to healthcare seeking and commonly sought health and health systems information from informal Thai networks made up of friends and acquaintances. Thais who had been in Norway for many years were often identified as key people one should approach. Some participants also sought information directly from their GP, from websites or from their Norwegian partner.

“Mostly I asked my Thai friends. We would provide suggestions for each other. We could not talk with anyone else. I do not have a family here. I have Thai people that I can talk to … .. My Thai friends (are the people) from whom I seek health information.” Isra (30-39), 3 years in Norway

Almost a third of the participants [5], all of whom had been in Norway for more than 3 years, indicated that they would make an appointment and go directly to see their GP to address their health concerns. Several Norwegian husbands, had helped sign women up for the GP scheme. Bringing informal interpreters, such as family members including children, friends or a social worker, to healthcare appointments also helped women to access care.

However, for those with limited Norwegian language proficiency, getting appointments and utilizing healthcare often involved complex translation strategies. Chaisee, a masseuse in her fifties, located health information on the internet, translated it into Norwegian using Google Translate, and booked doctor appointments with the assistance of a Norwegian friend who she hired to do small jobs.

Participants reflected that having access to an official translator would help them to more easily utilize Norwegian healthcare. Sharing information about the Norwegian health system with the Thai community, through the public health team that visits the massage shops or by utilizing other communication channels, was also identified by participants as a potential intervention to improve access to healthcare.

#### Seeking other solutions

Some participants decided to self-treat for their health needs and others went abroad to seek medical care.

For minor health concerns, like allergies, the common cold and pain relief, participants sought over the counter medication from pharmacies in Norway. Often, they also brought medicine, including pain medication, birth control pills and antibiotics, from Thailand.

“The doctor asked me do I want to take some medicine. I rejected. I do not want to take medicine. I have my own Thai drugs. The medicine is for muscle pain relief. These kinds of medicine from Thailand are stronger than the same drugs available in Norway.” Hom (50-59), 18 years in Norway

In self-treating, Hom described that some women consult their social network in Norway when they need pain medication and will get prescription drugs from a friend. “There is a friend who works physically hard, another friend replied on Facebook that she has some medicine for kidneys dialysis. Would you like to take it?”

Birth control pills, are available over the counter at pharmacies in Thailand, and Phet [20–29] received some from a friend, “Sometimes, my friends go to Thailand. They buy enough packages for two years. Some friends give me pills for 6 months, and others might give me pills for one year.”

While working in Norway, some women went abroad to Sweden and Thailand to seek medical care. Several participants with Swedish citizenship indicated that they travelled back and forth frequently between Norway and Sweden and could easily go to see their GP on one of these trips.

Language was the primary reason that Thai masseuses sought healthcare in Thailand. Sanit (40–49), lived in Norway for 10 years but gets a health check-up yearly during her visits to Thailand due to ease of language comprehension. In some cases, therapies offered in Thailand outside of the public healthcare system are not available in Norway. Buppha (50–59), had torn muscles as a result of an occupational health injury from doing deep tissue massage and originally sought healthcare in Norway from her GP and a chiropractor before deciding to borrow money from a friend and go to Thailand for intensive massage therapy.

## Discussion

Our research is specific to Thai immigrant masseuses, however we found that participants experiences accessing healthcare were more influenced by their experience as immigrants in Norway than their occupation as masseuses. The lack of influence of occupation on access to healthcare is likely to be partly due to the existence of a tax funded national healthcare system in Norway which offers universal health coverage to all citizens and residents. Norwegian employers have basically no role in financing health care for their employees. In contrast, a study with masseuses in the United States found that ability to pay and lack of insurance were barriers to accessing care [16]. Access to care in the Norwegian health system is only to a very limited extent based on ability to pay, while private insurance and out-of-pocket payments play a larger role in the US system. All migrants that reside regularly in Norway for more than 6 months are entitled to public tax funded health care under the same conditions as Norwegians. Study participants who were marriage immigrants or migrants with regular status were thus entitled to public healthcare, but still experienced difficulties related to their positionality as female Thai immigrants.

Thai masseuses in this study regarded the Norwegian healthcare system as high quality, but faced difficulties accessing correct health service information, navigating the system and getting appropriate care mainly related to the intersection of language challenges and cultural values. Language remained a persistent challenge for Thai immigrants in our study and is a key barrier to healthcare access and utilization. Even women who had been in Norway for over a decade, had difficulty understanding their Norwegian speaking clinician. Strategies used to overcome language barriers were often elaborate and resource intensive, including hiring someone to translate, and risk creating parallel systems to those offered by the Norwegian healthcare system. Other studies from Norway and Sweden also identified language as a barrier to care for Thai and other migrant groups [6, 7, 13]. Officially non-Norwegian native speakers are entitled to interpreters, but in practice women explained that they are not available or are only reserved for particularly serious cases. It is unclear whether this is a result of miscommunication of the policy from the care providers to the patient or if there is some unofficial rationing of interpreters by hospitals and clinics. Other studies with immigrants in Norway have documented difficulties with the implementation of the interpreter policy and an individual’s ability to get one when needed [6, 7, 27].

Our results that Thai marriage migrants who present with their Norwegian partner, are rarely offered an interpreter mirror a study from Sweden in which it was assumed that Thai women have “high social capital” as a result of their Swedish partner and are therefore not offered interpretation [13]. Our findings question the assumption that these women, as marriage immigrants with Norwegian partners, have better access to healthcare through assistance from their spouse, than other immigrant populations in Norway. Furthermore, we found some pervasive effects of this assumption when the relationship among partners was not good. Concerns we identified about the local spouses’ language capacity to translate into Thai are also documented in other studies in Norway and Sweden [6, 13]. From discussions with community stakeholders, we understand that Norwegian husbands often speak only limited Thai, or none at all, which would negate the value of having them interpret.

Beyond the potential limited effectiveness of local husbands as interpreters, lie additional considerations about the robustness of healthcare entitlements when individuals have to access them through someone else. Ultimately, entitlements are less robust, when you access them through someone who assists out of good will. In the case of an abusive husband, this means that the Thai woman has to seek assistance from someone who has caused them physical or psychological harm when accessing health care. This situation is counter to the principle of equitable and fair access which remains a cornerstone of the Norwegian public health system [28].

It appears that there is a threshold of difficulty beyond which immigrants will decide not to seek healthcare in Norway and instead find other alternatives, such as going to another country. Returning to one’s home country to access healthcare as described in this study is not unique to Thais and has also been documented among Polish migrants in Norway among others [7]. We anticipate that different cultural groups may have varying levels of tolerance for difficulty before they ultimately decide to opt-out and we expect that different economic possibilities to travel to their home countries could also influence this decision. This choice may also be related to their expectation of health services based on their past experience, as well as the cultural appropriateness of arguing for one’s rights. For Thai women, it is often inappropriate to complain and considered preferable to take care of one’s self, therefore the threshold before deciding not to seek care from the Norwegian healthcare system may be quite low comparative to other groups. Our study contributes to the literature on the health of Thai migrants by identifying cultural values which influence health seeking behaviour.

A previous Norwegian study indicated that immigrants use primary healthcare less than the general Norwegian population but it is uncertain whether this is due to good health or difficulty accessing care [29]. Our results suggest that there are substantial challenges for Thai marriage migrants who are seeking care. More research, including quantitative studies, looking at the socio-economic conditions of Thai marriage migrants are warranted to better understand the health needs of Thai women in Norway and other countries. Comparative studies including female migrants from other countries may be especially useful in investigating commonalities among groups, with the aim of developing recommendations for a diversity friendly health system.

As countries like Norway strive to have UHC, it is important not to lose sight of equity considerations and the need to develop immigrant sensitive health systems. From a practice perspective, there are small changes that can be made to the Norwegian healthcare system to make it easier for this population to get care. Outreach through Thai networks, could improve Thai migrants’ knowledge of the Norwegian healthcare system. The strength of Thai networks as information sources has been documented by another Norwegian study and a network approach to disseminate information appears warranted [6, 30]. Providing more health systems information in the fifty-hour training course that marriage migrants take when they arrive in Norway could also help reach this group. Challenges with accessing primary care, could be eased by systematically offering Thai women an interpreter, irrespective of whether their Norwegian husband attends the appointment with them. Internationally, use of interpretation services has been found to be an effective intervention to improve access to healthcare [31–33]. Having one’s partner translate can perpetuate unequal gender power dynamics and is often inappropriate given spouses’ inability to correctly interpret medical terms into Thai language. In offering a professional interpreter, clinicians should emphasize that the service is freely available, that the request does not place a burden on the care provider and that the waiting time for interpretation services will be minimized.

Another option, which may require additional health system adaptation, is to recruit Thai patient navigators to assist with interpretation and system navigation. In other contexts, cultural navigators and community health workers have been effective in improving access to care for minority populations [32]. Beyond improving communication, GPs mapping of immigrants’ backgrounds and migration trajectories as suggested by Goth et al. can help clinicians to provide appropriate and contextually relevant care [34]. Our study supports the importance of mapping as we show that Thai women may have different health care seeking experiences and options depending on whether they immigrated first to another European country before coming to Norway.

## Limitations

In interpreting this study, it is important to emphasize that the results cannot be generalized to all Thai immigrants living in Norway. By interacting with a public health team, our participants may have better access to healthcare than Thai migrants who are not working as masseuses. Our sample was an urban population, while many Thai migrants live in rural areas and may have different challenges accessing care including transportation. In addition, compared to other studies with Thai marriage migrants our group had lower levels of education, which may influence health seeking behavior [6, 13]. Future studies may wish to investigate the experience of rural Thai migrants.

## Conclusion

Despite having the same entitlements to healthcare as Norwegian citizens, Thai marriage migrants face navigation challenges and experience difficulties accessing healthcare, particularly due to pervasive language barriers. A significant gap exists between the official policy that free interpreters should be provided and the experiences reported. Action is required to ensure equitable access to healthcare for Thai immigrant women in Norway.

In line with official policy, health personnel should offer professional interpreters and not rely on Norwegian spouses to translate. Thai immigrants’ knowledge and ability to navigate the Norwegian healthcare system may also be further promoted by use of community health workers and outreach through Thai networks.

## Supplementary information

**Additional file 1:** Interview guide for female migrants working in as masseuses.

## Data Availability

The data we collected contains personally identifying information. To protect participant confidentiality we cannot share the data.

## Abbreviations

GP: General practitioner
