# Supplementary material for: Accessing public healthcare in Oslo, Norway: the experiences of Thai immigrant masseuses
Source: BMC Health Serv Res. 2019 Oct 21;19:722. doi: 10.1186/s12913-019-4560-9 (PMC6805446; doi:10.1186/s12913-019-4560-9)
Supplement: Supplementary file 1 — Additional file 1: Interview guide for female migrants working in as masseuses. [file 12913_2019_4560_MOESM1_ESM.pdf]

Interview guide for female migrants working in as masseuses

Intro

The purpose of this interview is to help us better understand your experiences locating and accessing primary health services in Norway. "Primary care is the provision of integrated, accessible health care services by clinicians who are responsible for addressing a large majority of personal health care needs, developing a sustained partnership with patients, and practicing in the context of family and community."(1) One role of primary health care is the initial management of urgent conditions and then referral to a specialist. Primary healthcare providers also give care for preventative and non-urgent conditions.

We will ask some questions about how you access care for your reproductive and sexual health needs. Reproductive health is one's "physical, mental and social well-being…. relating to the reproductive system".(2)

We are also interested to learn more about your experience as a migrant in Norway.

Migration trajectory

Where is your hometown? What was your occupation in Thailand? Where were you living before you moved here to Norway?

Work in Norway

In the last 12 months, have you worked as a masseuse in Oslo? Do you have more than one job?

Registered GP Scheme

Do you have a general practitioner in Norway?

- If yes – Can you describe how you signed up for this scheme?
- If no – Are you aware of the GP scheme?

Access to healthcare (general)

Where do you go for your non-urgent healthcare concerns?

Can you describe your most recent experience accessing healthcare in Norway?

Health system literacy and navigation

When seeking healthcare in Norway have you experienced any challenges (barriers)?

- Probes: language, cost, etc.

If you needed to access a healthcare service, where would you seek information?

- Probes: Friends, family, websites, organizations, etc.

Situational question (access strategies)

Imagine that you meet Nok, a Thai woman who recently married a Norwegian man and immigrated to Norway 8 months ago. Nok speaks very little Norwegian and has asked you where she can access birth control pills. What would you recommend that she do?

Primary care for reproductive health

Contraception

Are you currently using contraception?

- If yes – Could you describe the method (barrier, pill, long acting)?
- If yes – Can you describe how you accessed this contraception?
- If no – Can you describe why you are not using contraception?

Preventative

Have you been offered cervical cancer screening in Norway within the last three years?
Did you take the pap test for cervical cancer screening within the last three years?

Have you been screened for sexually transmitted infections (STIs) in the past 12 months? If you wanted to be screened where would you go?

Health needs

Do you have any unmet health needs?

Health system improvements

Can you identify any improvements that could make it easier for you to access healthcare?

Coping strategies for stress

Do you experience stress in your daily life in Norway? How do you cope with this stress?
Have you ever sought help for your mental health?

Reference

1. Institute of Medicine. Defining Primary Care: An Interim Report. Committee on the Future of Primary care, Division of Health Care Services, IOM.; 1994.

2. Glasier A, Gülmezoglu AM, Schmid GP, Moreno CG, Look PFV. Sexual and reproductive health: a matter of life and death. The Lancet. 2006;368(9547):1595–607.
